# Surface Structures Formed by a Copper(II) Complex of Alkyl-Derivatized Indigo

**DOI:** 10.3390/ma9100837

**Published:** 2016-10-15

**Authors:** Akinori Honda, Keisuke Noda, Yoshinori Tamaki, Kazuo Miyamura

**Affiliations:** 1 Department of Chemistry, Faculty of Science, Tokyo University of Science, 1-3 Kagurazaka, Shinjuku-ku, Tokyo 162-8601, Japan; keisuke.noda.ph@hitachi.com (K.N.); tamaki_y@dent.meikai.ac.jp (Y.T.); 2 Hitachi, Ltd., Power Generation Plant & Grid Control Systems Engineering Department, Control System Platform Division, 5-2-1 Omika-cho, Hitachi-shi, Ibaraki 319-1293, Japan; 3 School of Dentistry, Faculty of Dentistry, Meikai University, 1-1 Keyakidai, Sakado-shi, Saitama 350-0283, Japan

**Keywords:** dye, scanning tunneling microscope, alkyl chain, indigo, copper, mordanting

## Abstract

Assembled structures of dyes have great influence on their coloring function. For example, metal ions added in the dyeing process are known to prevent fading of color. Thus, we have investigated the influence of an addition of copper(II) ion on the surface structure of alkyl-derivatized indigo. Scanning tunneling microscope (STM) analysis revealed that the copper(II) complexes of indigo formed orderly lamellar structures on a HOPG substrate. These lamellar structures of the complexes are found to be more stable than those of alkyl-derivatized indigos alone. Furthermore, 2D chirality was observed.

## 1. Introduction

Dyes are colored substances capable of absorbing certain wavelengths of light intensively. Many of the dyes have a large π-conjugated system, and the color of dyes is highly influenced by the electronic state of the system. In consequence, assembled structural changes often induce the change in the electronic state of the π-conjugated system and result in color variations. Color changes via polymorphism, phase transition, etc. have been reported elsewhere [1,2,3]. Thus, an understanding of how molecular assembly of dyes is constructed is necessary for the design of a coloring function. Dyes often have a planar structure due to the large π-conjugated system, resulting in an affinity to the substrate onto which they adsorb. This is why the assembled structures formed by the dyes on substrates have been analyzed in detail [4,5,6]. A scanning tunneling microscope (STM) [7] is an instrument suitable for imaging the surface structures on a substrate, because STM is capable of resolving the surface structures at the atomic level, and the ordered molecular images of self-assembled structure of dyes can be observed [8,9,10,11,12].

**Figure 1:**
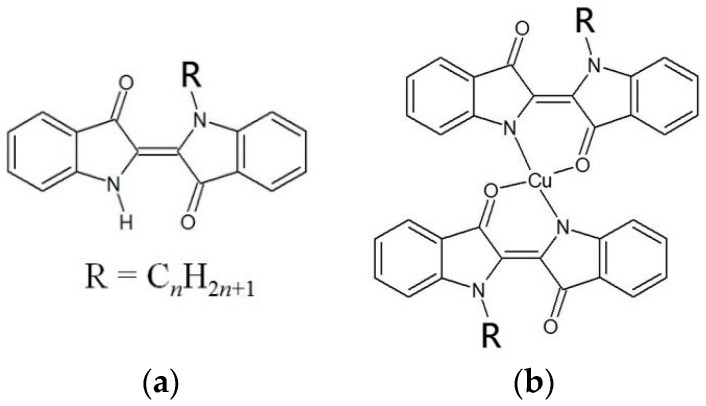
Chemical structures of (**a**) C*n*IND and (**b**) [Cu(C*n*IND)_2_].

Indigo dye is an organic dye exhibiting a distinct blue color, used for dyeing jeans blue. The indigo molecule has a planar structure due to the presence of a π-conjugated system and thus tends to assemble on a HOPG or metallic substrate. In our previous study, the surface structures of alkyl-derivatized indigo (C*n*IND, *n* = alkyl chains length) shown in Figure 1a were analyzed via STM, which revealed that C*n*IND formed characteristic monolayer structures on HOPG [13]. In this study, we synthesized [Cu(C*n*IND)_2_], the copper(II) complex of C*n*IND, shown in Figure 1b and observed the surface structures formed by [Cu(C*n*IND)_2_] using STM. This investigation was carried out to understand the influence of the addition of metal ions, which often stabilizes the adsorption of dyes on the substrate. This phenomenon is known as mordanting. Mordanting [14,15] is the process of adding metal ions (Cu, Fe, etc.) in a dyeing process, so as to prevent the color loss of a dyeing object. The role of metal ions is estimated as filling the possible space between dye molecules and to stabilize the adsorption of the dye molecules onto a dyeing object. By comparing the surface structures of C*n*IND and those of [Cu(C*n*IND)_2_], the mechanism of stabilization via mordanting may be found. In addition, [Cu(C*n*IND)_2_] exhibited a unique structure exhibiting 2D and 3D chirality, which is described later.

## 2. Results and Discussion

### 2.1. STM Images of [Cu(C16IND)_2_] on HOPG

**Figure 2:**
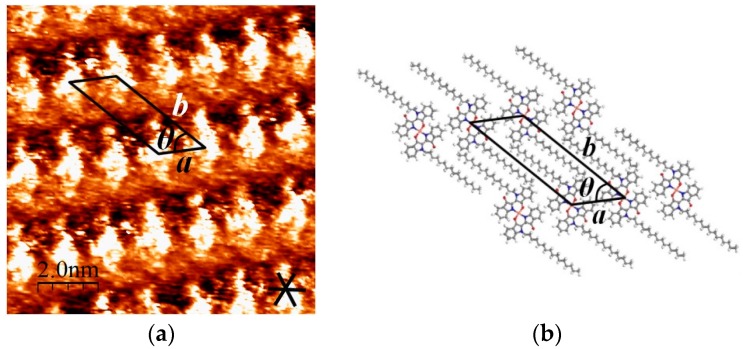
(**a**) Scanning tunneling microscope (STM) image of self-assembled [Cu(C16IND)_2_] at a HOPG/o-dichlorobenzene interface. Image area: 10 × 10 nm^2^; V_set_ = 1.02 V, I_set_ = 981 pA; (**b**) structural model for [Cu(C16IND)_2_]; unit cell parameters: a = 1.5 nm, b = 3.6 nm, θ = 48°. The unit cell is taken so as to include the alkyl chains of a C16IND.

We successfully observed the structures of a self-assembled monolayer formed by [Cu(C16IND)_2_] on a HOPG surface using STM. The obtained STM image and structural model of the monolayer are shown in Figure 2a,b, respectively. [Cu(C16IND)_2_] molecules formed a lamellar structure, with complex moieties imaged as bright spots in the STM image, since π-conjugation is generally more conductive than aliphatic alkyl chains. Alkyl chains are assumed to align in the darker regions between the bright spots of an STM image and are interdigitated as shown in Figure 2b. Interdigitated alkyl chains are known to have large van der Waals interactions between alkyl chains and the HOPG substrate, seeming to play a great role in controlling the surface structures of [Cu(C16IND)_2_].

### 2.2. Comparison between [Cu(C16IND)_2_] and C16IND

**Figure A1:**
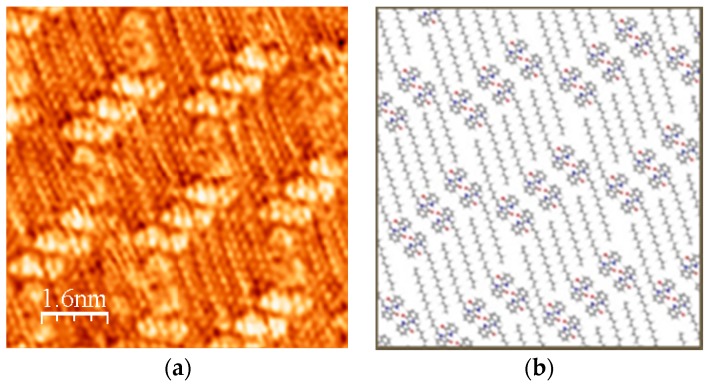
Dislocated structure formed by C16IND: (**a**) STM image; (**b**) structural model.

In the previous study, the self-assembled structures on HOPG of C16IND were analyzed via STM [13]. C16IND molecules formed a dimer via hydrogen bonds, and alkyl chains were interdigitated via van der Waals interactions. In addition, C16IND molecules formed a dislocation structure at every three molecules, as shown in Figure A1. This dislocation along the molecular lamella axis derives from the matching between the alkyl chains and the HOPG substrate lattice [16,17].

**Figure 3:**
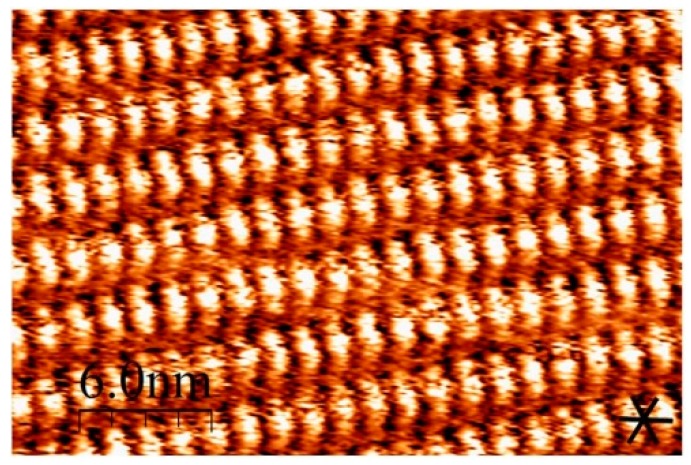
Larger scale STM image of [Cu(C16IND)_2_]. Image area: 30 × 20 nm^2^.

Compared with C16IND, the copper complex [Cu(C16IND)_2_] formed an orderly structure, and the dislocation was not observed (Figure 3). The ordered structure of [Cu(C16IND)_2_] indicates that adding copper ions to C16IND results in a stabilization of the adsorbed structures. The result may suggest the effect of mordanting, which is to add metal ions in a dyeing process.

### 2.3. 2D Chirality of [Cu(C16IND)_2_]

**Figure 4:**
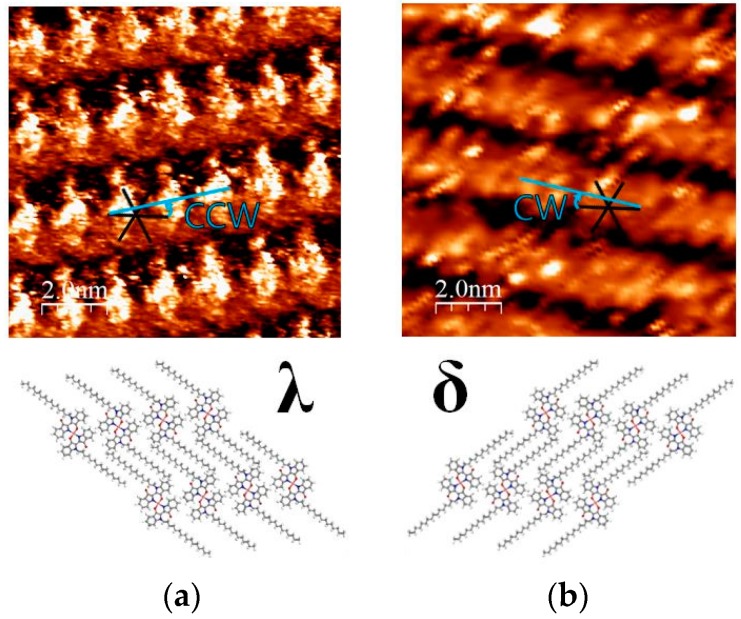
2D chirality of [Cu(C16IND)_2_]: (**a**) λ-structure; (**b**) δ-structure.

“2D chirality” of the adsorbed molecules on the substrate has been intensively studied [18,19,20]. When a prochiral molecule adsorbs onto a substrate, the molecular inversion is restricted and the adsorbed molecules can be distinguished as enantiomers (λ and δ in Figure 4). 

[Cu(C16IND)_2_] on the HOPG surface exhibits distinguishable 2D chirality, as shown in Figure 4. In the λ-structure shown in Figure 4a, molecular columns aligned counter-clockwise (CCW) versus the HOPG substrate lattice. The angle between them is approximately 10°. On the other hand, Figure 4b shows a δ-structure, and the molecules were aligned by the same angle but in a clockwise (CW) orientation versus the HOPG lattice.

### 2.4. STM Images of [Cu(C18IND)_2_] and [Cu(C20IND)_2_] on HOPG

**Figure 5:**
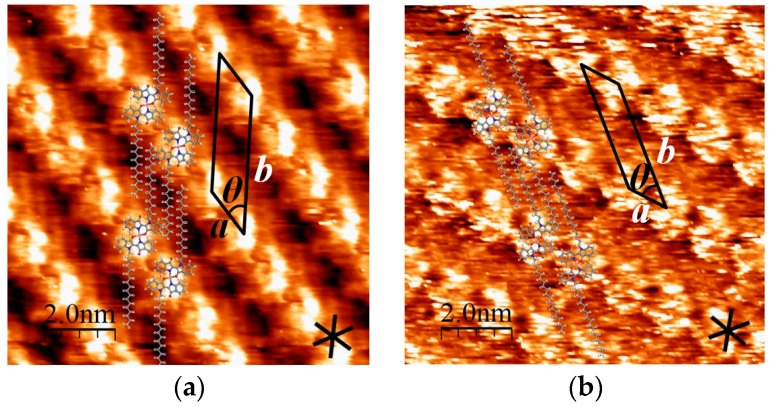
(**a**) STM image and structural model of self-assembled [Cu(C18IND)_2_] at a HOPG/o-dichlorobenzene interface. Image area: 10 × 10 nm^2^; V_set_ = 1.41 V, I_set_ = 697 pA; unit cell parameters: a = 1.5 nm, b = 3.7 nm, θ = 41°; (**b**) STM image and structural model of [Cu(C20IND)_2_]. Image area: 10 × 10 nm^2^; V_set_ = 1.41 V, I_set_ = 697 pA; unit cell parameters: a = 1.4 nm, b = 4.0 nm, θ = 40°.

Alkyl chains lengths are known to influence the self-assembled structures of alkyl-derivatives [21,22,23,24,25,26]. Varying the chain lengths results in changing the intermolecular and molecule-substrate interactions, leading to a surface structural variation. Thus, we investigated surface structures formed by [Cu(C18IND)_2_] and [Cu(C20IND)_2_] with longer alkyl chains than [Cu(C16IND)_2_]. As a result, [Cu(C18IND)_2_] and [Cu(C20IND)_2_] molecules were found to form basically similar structures to [Cu(C16IND)_2_]. These molecules formed a similar lamellar structure, and alkyl chains were interdigitated as shown in Figure 5a,b. The alkyl chains of [Cu(C18IND)_2_] seem to be aligned along the HOPG substrate lattice, suggesting that the surface structure matches well with the HOPG lattice and becomes stable.

### 2.5. The Effect of 3D Chirality of [Cu(C20IND)_2_] to 2D Self-Assembly

**Figure 6:**
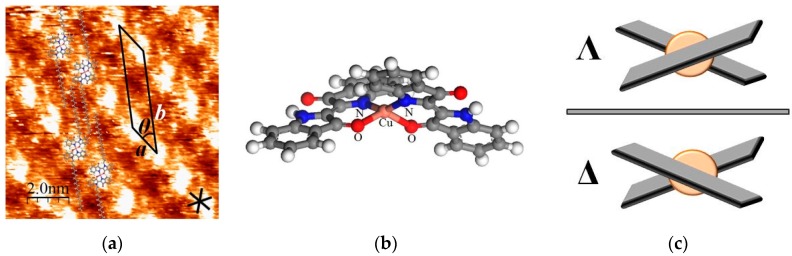
(**a**) STM image and structural model of type 2 structure formed by [Cu(C20IND)_2_]. Image area: 10 × 10 nm^2^; V_set_ = 1.41 V, I_set_ = 697 pA; unit cell parameters: a = 1.7 nm, b = 4.8 nm, θ = 34°; (**b**) twisted coordination model of [Cu(IND)_2_]; (**c**) 3D chiral model of [Cu(IND)_2_].

[Cu(C20IND)_2_] also exhibited a different surface structure (namely type 2 structure shown in Figure 6a) from that described in Figure 5b (type 1 structure). The 3D chirality of [Cu(IND)_2_] shown in Figure 6c seems to cause the molecules to form these different structures. The coordination structure of [Cu(IND)_2_] was calculated using Materials Studio 7.0 software [27] (Accelrys Inc., San Diego, CA, USA), in which the density functional theory (DFT) calculations were performed to optimize the structure using generalized gradient approximation (GGA) proposed by Perdew et al. (PBE) [28]. Figure 6b shows the calculated structure, and it revealed that [Cu(IND)_2_] adopts the twisted square planar geometry due to the steric repulsion between coordinated indigo moieties in a complex. Owing to the twisted structure, 3D chirality of [Cu(IND)_2_] appears as shown in Figure 6c. As a result, Λ-structure and Δ-structure can be distinguished.

Compared with the type 1 structure of [Cu(C20IND)_2_] shown in Figure 5b, the type 2 structure shown in Figure 6a has a longer b-axis. Since the b-axis is taken along the orientation of alkyl chains, the difference in the b-axis length suggests a variation in the assembled structure of alkyl chains. When the twisted complex adsorbs onto the HOPG substrate, a space between the complex and the substrate appears due to the non-planarity. Thus, the end group of alkyl chains can enter into the space depending on the situation. In the type 1 structure, the short length of the b-axis indicates that the end group of alkyl chains is inserted into the space. On the other hand, this insertion is blocked by the phenyl groups of the indigo in the type 2 structure. Hence, the steric structure of [Cu(C20IND)_2_] molecules in the type 1 structure is different from that in the type 2 structure. In other words, the 3D chirality of [Cu(C20IND)_2_] induced a structural difference.

## 3. Materials and Methods

### 3.1. Materials and Identification

All chemicals including indigo were purchased from Tokyo Chemical Industry, Aldrich or Kanto Chemicals, and used without further purification. Elemental analyses were performed on a Perkin-Elmer 2400II CHN analyzer. IR spectra were measured on a JASCO FT/IR-4200 spectrophotometer using KBr pellets. UV-vis absorption spectra were measured on a JASCO V-750 spectrophotometer using chloroform as a solvent. The concentration of the sample for UV-vis absorption spectra was 1.0 × 10^−5^ mol·L^−1^.

### 3.2. Synthesis of the Copper(II) Complex of Hexadecyl-Indigo ([Cu(C16IND)_2_])

*N*-hexadecyl-indigo (C16IND) was synthesized by the scheme in a previous study [13]. A total of 3.0 mmol (1.5 g) of C16IND in 40 mL of *N*,*N*-dimethylformamide was added to a 200 mL recovery flask equipped with a magnetic stirring bar. After the addition of 1.5 mmol (0.27 g) of copper(II) acetate, the mixture was stirred and refluxed for 8 h. After the reflux, a blue suspension was obtained. The suspension was cooled to room temperature, and then filtrated under suction to remove the solvent. The copper(II) complex of hexadecyl-indigo: [Cu(C16IND)_2_] was obtained as blue crystals in ca. 25% yield. Elemental analysis, Calc: C, 74.28; H, 7.99; N, 5.41%, Found: C, 73.92; H, 8.29; N, 5.35%; IR (KBr): *ν* 1694 cm^−1^ (C=C), 1702 (C=O), 2848–2950 (C-H); UV-vis *λ*_max_ (*ε*): 299 nm (5.7 × 10^4^ L·mol^−1^·cm^−1^), 350 nm (2.1 × 10^4^ L·mol^−1^·cm^−1^), 651 nm (2.1 × 10^4^ L·mol^−1^·cm^−1^), 752 nm (1.1 × 10^4^ L·mol^−1^·cm^−1^, shoulder peak).

### 3.3. Synthesis of the Copper(II) Complexes of Octadecyl-Indigo ([Cu(C18IND)_2_]) and Icosyl-Indigo ([Cu(C20IND)_2_])

We synthesized [Cu(C18IND)_2_] and [Cu(C20IND)_2_] with the same scheme as that with which [Cu(C16IND)_2_] was synthesized described above, using C18IND and C20IND respectively instead of C16IND. [Cu(C18IND)_2_]: Elemental analysis, Calc: C, 74.86; H, 8.31; N, 5.14%, Found: C, 74.84; H, 8.17; N, 5.39%; IR (KBr): *ν* 1695 cm^−1^ (C=C), 1702 (C=O), 2847–2960 (C-H); UV-vis *λ*_max_ (*ε*): 299 nm (5.5 × 10^4^ L·mol^−1^·cm^−1^), 348 nm (2.1 × 10^4^ L·mol^−1^·cm^−1^), 650 nm (2.2 × 10^4^ L·mol^−1^·cm^−1^), 752 nm (9.5 × 10^3^ L·mol^−1^·cm^−1^, shoulder peak). [Cu(C20IND)_2_]: Elemental analysis, Calc: C, 75.39; H, 8.61; N, 4.88%, Found: C, 75.58; H, 8.74; N, 4.71%; IR (KBr): *ν* 1695 cm^−1^ (C=C), 1702 (C=O), 2847–2948 (C-H); UV-vis *λ*_max_ (*ε*): 299 nm (5.7 × 10^4^ L·mol^−1^·cm^−1^), 349 nm (2.2 × 10^4^ L·mol^−1^·cm^−1^), 650 nm (2.2 × 10^4^ L·mol^−1^·cm^−1^), 752 nm (1.1 × 10^4^ L·mol^−1^·cm^−1^, shoulder peak).

### 3.4. Scanning Tunneling Microscope (STM) Measurements

Surface structures formed by [Cu(C16IND)_2_], [Cu(C18IND)_2_], and [Cu(C20IND)_2_] on HOPG were observed via STM (Digital Instruments Co., Santa Barbara, CA, USA: Nanoscope II/E). STM scans were performed at the *o*-dichlorobenzene/HOPG interface, at room temperature and under ambient pressure. STM tips were prepared via electrochemical etching from Tungsten wire (0.25 mmφ, Nilaco, Tokyo, Japan). The details of the measuring conditions are as follows: each solution of [Cu(C16IND)_2_], [Cu(C18IND)_2_], and [Cu(C20IND)_2_] in *o*-dichlorobenzene was prepared in a concentration of 1.0 mmol·L^−1^. A drop of this *o*-dichlorobenzene solution was applied onto a freshly cleaved surface of HOPG (ZYB grade, NT-MDT, Moscow, Russia). Then, STM scans were performed at the solution/HOPG interface in constant current mode. The STM images were corrected and analyzed via WSxM 5.0 software [29] (Nanotec, Madrid, Spain), using the HOPG substrate images recorded for calibration. Structural models fitting in the STM images were made by the Materials Studio 7.0 software (Accelrys Inc., San Diego, CA, USA).

## 4. Conclusions

We investigated the two-dimensional self-assembled structures formed by copper(II) complexes of alkyl-derivatized indigo ([Cu(C*n*IND)_2_]). STM images revealed that the assembled structures of the complexes were more stable than those of alkyl-derivatized indigos alone, suggesting the effect of mordanting. [Cu(C*n*IND)_2_] formed orderly lamellar structures on a HOPG substrate, and their alkyl chains were interdigitated. In the STM study of [Cu(C16IND)_2_], 2D chirality was observed; *λ*-structure and *δ*-structure were distinguished by molecular orientation. In the STM study of [Cu(C20IND)_2_], two types of structures with different lengths of *b*-axis were observed. The difference between these structures was found to derive from the 3D chirality of [Cu(C20IND)_2_]. In summary, [Cu(C*n*IND)_2_] formed orderly surface structures involving 2D chirality, and these surface structures were altered by 3D chirality.

## Abbreviations

The following abbreviations are used in this manuscript:

STM	scanning tunneling microscope
HOPG	high orientated pyrolytic graphite
2D	two-dimensional
3D	three-dimensional

## Appendix A

In the previous study, a dislocated structure of C16IND was observed as shown in Figure A1.

## References

[1] Kim H., Chang J.Y. (2014). Reversible Thermochromic Polymer Film Embedded with Fluorescent Organogel Nanofibers. Langmuir.

[2] Imai Y., Kinuta T., Nagasaki K., Harada T., Sato T., Tajima N., Sasaki Y., Kuroda R., Matsubara Y. (2009). Conformational and color polymorphism of achiral 2-methyl-3-(2-naphthalenylthio)-1,4-naphthalenedio-ne. Cryst. Eng. Comm..

[3] Lee J.H., Naumov P., Chung I.H., Lee S.C. (2011). Solid-State Thermochromism and Phase Transitions of Charge Transfer 1,3-Diamino-4,6-dinitrobenzene Dyes. J. Phys. Chem. A.

[4] Ono S.S., Yao H., Matsuoka O., Kawabata R., Kitamura N., Yamamoto S. (1999). Anisotropic Growth of J Aggregates of Pseudoisocyanine Dye at a Mica/Solution Interface Revealed by AFM and Polarization Absorption Measurements. J. Phys. Chem. B.

[5] Nguyen N.D., Zhang G., Lu J., Sherman A.E., Fraser C.L. (2011). Alkyl chain length effects on solid-state difluoroboron β-diketonate mechanochromic luminescence. J. Mater. Chem..

[6] Kuroda S. (2004). J-aggregation and its characterization in Langmuir–Blodgett films of merocyanine dyes. Adv. Colloid Interface Sci..

[7] Binnig G., Rohrer H., Gerber C., Weibel E. (1982). Surface Studies by Scanning Tunneling Microscopy. Phys. Rev. Lett..

[8] Thalacker C., Miura A., de Feyter S., de Schryver F.C., Würthner F. (2005). Hydrogen bond directed self-assembly of core-substituted naphthalene bisimides with melamines in solution and at the graphite interface. Org. Biomol. Chem..

[9] Wang D., Wan L.-J., Wang C., Bai C.-L. (2002). In Situ STM Evidence for Adsorption of Rhodamine B in Solution. J. Phys. Chem. B.

[10] Jaroch T., Maranda-Niedbala A., Kotwica K., Wamil D., Bujak P., Pron A., Nowakowski R. (2015). Self-assembly of tetraalkoxydinaphthophenazines in monolayers on HOPG by scanning tunneling microscopy. Surface Sci..

[11] Stawasz M.E., Sampson D.L., Parkinson B.A. (2000). Scanning Tunneling Microscopy Investigation of the Ordered Structures of Dialkylamino Hydroxylated Squaraines Adsorbed on Highly Oriented Pyrolytic Graphite. Langmuir.

[12] Kawasaki M., Sato T., Yoshimoto T. (2000). Controlled Layering of Two-Dimensional J-Aggregate of Anionic Cyanine Dye on Self-Assembled Cysteamine Monolayer on Au(111). Langmuir.

[13] Urano K., Ohno T., Tomono K., Miyamura K. (2013). Observation of Dynamic Behavior of Self-Assembled *N*-Icosyl-Substituted Indigo by STM. Bull. Chem. Soc. Jpn..

[14] Guzel B., Akgerman A. (2000). Mordant dyeing of wool by supercritical processing. J. Supercrit. Fluids.

[15] Moiz A., Ahmed M.A., Kausar N., Ahmed K., Sohail M. (2010). Study the effect of metal ion on wool fabric dyeing with tea as natural dye. J. Saudi Chem. Soc..

[16] Mali K.S., Lava K., Binnemans K., de Feyter S. (2010). Hydrogen Bonding Versus van der Waals Interactions: Competitive Influence of Noncovalent Interactions on 2D Self-Assembly at the Liquid–Solid Interface. Chem. Eur. J..

[17] Tamaki Y., Muto K., Miyamura K. (2013). Odd-Even Effect in the Surface Structure of Alkyloxy-Substituted Anthraquinone on HOPG Observed by Scanning Tunneling Microscope. Bull. Chem. Soc. Jpn..

[18] Vidal F., Delvigne E., Stepanow S., Lin N., Barth J.V., Kern K. (2005). Chiral Phase Transition in Two-Dimensional Supramolecular Assemblies of Prochiral Molecules. J. Am. Chem. Soc..

[19] Böhringer M., Schneider W.-D., Berndt R. (2000). Real Space Observation of a Chiral Phase Transition in a Two-Dimensional Organic Layer. Angew. Chem. Int. Ed..

[20] Honda A., Tamaki Y., Miyamura K. (2015). The Effects of Noncovalent Interactions on Surface Structures Formed by Diketopyrrolopyrrole Pigment and Its Alkyl-Derivatives on HOPG Substrate. Bull. Chem. Soc. Jpn..

[21] Miyake K., Hori Y., Ikeda T., Asakawa M., Shimizu T., Sasaki S. (2008). Alkyl Chain Length Dependence of the Self-Organized Structure of Alkyl-Substituted Phthalocyanines. Langmuir.

[22] De Feyter S., Larsson M., Schuurmans N., Verkuijl B., Zoriniants G., Gesquière A., Abdel-Mottaleb M.M., van Esch J., Feringa B.L., van Stam J. (2003). Supramolecular Control of Two-Dimensional Phase Behavior. Chem. Eur. J..

[23] Chen Q., Yan H.-J., Yan C.-J., Pan G.-B., Wan L.-J., Wen G.-Y., Zhang D.-Q. (2008). STM investigation of the dependence of alkane and alkane (C_18_H_38_, C_19_H_40_) derivatives self-assembly on molecular chemical structure on HOPG surface. Surface Sci..

[24] Mamdouh W., Uji-i H., Ladislaw J.S., Dulcey A.E., Percec V., de Schryver F.C., de Feyter S. (2006). Solvent Controlled Self-Assembly at the Liquid-Solid Interface Revealed by STM. J. Am. Chem. Soc..

[25] Hibino M., Sumi A., Tsuchiya H., Hatta I. (1998). Microscopic Origin of the Odd-Even Effect in Monolayer of Fatty Acids Formed on a Graphite Surface by Scanning Tunneling Microscopy. J. Phys. Chem. B.

[26] Tahara K., Furukawa S., Uji-I H., Uchino T., Ichikawa T., Zhang J., Mamdouh W., Sonoda M., de Schryver F.C., de Feyter S., Tobe Y. (2006). Two-Dimensional Porous Molecular Networks of Dehydrobenzo[12]annulene Derivatives via Alkyl Chain Interdigitation. J. Am. Chem. Soc..

[27] Accelrys Inc. (2013). Materials Studio.

[28] Perdew J.P., Burke K., Ernzerhof M. (1996). Generalized Gradient Approximation Made Simple. Phys. Rev. Lett..

[29] Horcas I., Fernández R., Gómez-Rodríguez J.M., Colchero J., Gómez-Herrero J., Baro A.M. (2007). WSXM: A software for scanning probe microscopy and a tool for nanotechnology. Rev. Sci. Instrum..

